# Challenges and opportunities for strengthening South American responses to insecticide resistance in disease vectors: Lessons from the WINSA workshop in Bogotá, Colombia

**DOI:** 10.1371/journal.pntd.0014809

**Published:** 2026-10-30

**Authors:** Ademir J. Martins, Martha Ahumada, Tashanna S. Bowman, Olga Lucia Cabrera, Kartika Doerdjan-Ramoutar, Luciana dos Santos Dias, Jean-Bernard Duchemin, Stephane Duchon, Luisa Figueroa, Adriana E. Flores, Christian R. González, Nilsa González-Brítez, Laura Harburguer, Audrey Lenhart, José Bento Pereira Lima, Ronald Lopez, Diego Morales, Martha L. Quinones, Miriam Palomino Salcedo, Gabriela Willat, Vincent Corbel

**Affiliations:** 1 Laboratório de Biologia, Controle e Vigilância de Insetos Vetores (LBCVIV), Instituto Oswaldo Cruz (IOC), FIOCRUZ, Rio de Janeiro, Brasil; 2 Grupo de Entomología, Instituto Nacional de Salud, Bogotá, Colombia; 3 Vector Control Services, Ministry of Health, Georgetown, Guyana; 4 Anton de Kom University of Suriname (AdeKUS), Leysweg, Paramaribo, Suriname; 5 Unité d’Entomologie Médicale, Institut Pasteur de la Guyane (IPG), Cayenne, French Guiana, France; 6 Arboviruses and Insect Vectors (AIV), Institut Pasteur, Paris, France; 7 Institut de Recherche pour le Développement (IRD), MIVEGEC, CNRS, IRD, Université de Montpellier, Montpellier, France; 8 Centro de Estudios de Enfermedades Endémicas y Salud Ambiental, SAIAE-MPPS, Maracay, Aragua, Venezuela; 9 Universidad de Carabobo, Naguanagua, Carabobo, Venezuela; 10 Laboratorio de Entomología Médica, Facultad de Ciencias Biológicas, Universidad Autónoma de Nuevo León, San Nicolás de los Garza, Nuevo León, México; 11 Instituto de Entomología, Facultad de Ciencias Básicas, Universidad Metropolitana de Ciencias de la Educación, Ñuñoa, Santiago, Chile; 12 Grupo Entomología Médica, Instituto de Investigaciones en Ciencias de la Salud, Universidad Nacional de Asunción, San Lorenzo, Paraguay; 13 Facultad de Ciencias Exactas y Naturales, Universidad Nacional de Asunción, San Lorenzo, Paraguay; 14 Centro de Investigaciones de Plagas e Insecticidas (CIPEIN-UNIDEF/CITEDEF/CONICET), Buenos Aires, Argentina; 15 Centers for Disease Control and Prevention (U.S. CDC), Atlanta, Georgia, United States of America; 16 Laboratorio de Entomología y Parasitología, Instituto Nacional de Laboratorios de Salud (INLASA), La Paz, Bolivia; 17 Unidad de Bienestar Animal, Secretaría de Salud, Gobierno Autónomo Descentralizado del Distrito Metropolitano (UBA-GADMQ), Quito, Ecuador; 18 Departamento de Salud Pública, Facultad de Medicina, Universidad Nacional de Colombia, Bogotá, Colombia; 19 Área de Vectores, Instituto Nacional de Salud (INS), Chorrillos, Lima, Peru; 20 Zoonosis y Vectores, Ministerio de Salud Pública, Montevideo, Uruguay; Centers for Disease Control and Prevention, Puerto Rico, UNITED STATES OF AMERICA

## Abstract

**Background:**

Vector-borne diseases remain a major and evolving public health challenge in South America, where arboviruses such as dengue, chikungunya, Zika, and malaria continue to expand in incidence and geographic distribution. Insecticides remain central to vector control programs; however, the rapid emergence and spread of insecticide resistance across multiple vector species increasingly threaten the effectiveness and sustainability of these interventions. In response to these challenges, the Worldwide Insecticide Resistance Network—South America (WINSA) organized a regional workshop in Bogotá, Colombia, in March 2026 to assess the current status of insecticide resistance, identify research priorities, and strengthen regional collaboration.

**Methods:**

The workshop brought together experts from 13 South American countries and territories representing academia, public health institutions, and international organizations. Sixteen presentations addressed the current status of insecticide resistance in major disease vectors, including mosquitoes and triatomines. Discussions covered surveillance systems, resistance mechanisms, molecular and genomic tools, operational challenges, and innovative vector control strategies.

**Results:**

The workshop highlighted widespread resistance to pyrethroids and organophosphates across multiple vector species in South America. High frequencies of knockdown resistance mutations (*kdr*) and evidence of metabolic resistance mechanisms were reported in several countries, particularly in the dengue vector *Aedes aegypti*. Emerging resistance to novel insecticide formulations was also identified. Participants emphasized major disparities in surveillance capacity, insufficient standardization of methodologies, and uneven geographical coverage across the region. An Integrated Vector Resistance management framework, integrating biological, molecular, genomic, and spatial surveillance approaches, was proposed to support evidence-based decision-making in vector control. Innovative approaches, including insecticide combinations, spatial repellents, and insecticidal paints, were presented as promising complementary tools for integrated vector management.

**Conclusions:**

Insecticide resistance represents a growing threat to the control of arboviral and malaria vectors in South America. Strengthening harmonized surveillance systems, integrating molecular and genomic tools, and improving regional coordination are critical priorities. WINSA provides a strategic scientific and technical platform to support collaborative research, capacity building, harmonized resistance surveillance, and the translation of resistance evidence into information that can support vector-control decision-making across the region.

## Introduction

Vector-borne diseases remain a major and evolving public health challenge in South America, where arboviruses such as dengue, chikungunya, and Zika continue to expand in both geographic range and incidence [1]. In Brazil, 2024 marked the largest dengue outbreak ever recorded, with more than 6.5 million reported cases and over 5,000 deaths, reflecting a major expansion of transmission into previously less affected regions [2]. At the same time, Oropouche virus has re-emerged and spread beyond its historical Amazonian range, with thousands of confirmed cases reported in Brazil in 2024 and continued transmission into 2025 [3]. In 2026, chikungunya cases were reported in Suriname, French Guiana, and northern Brazil, suggesting sustained regional transmission and an increased risk of large-scale epidemics [4]. Together, these overlapping epidemics highlight a concerning convergence of increased transmission intensity, geographic expansion, and the limited effectiveness of current vector control strategies [5].

Despite recent advances in vector control technologies, including *Wolbachia*-based approaches and sterile insect techniques [6], insecticides remain central to vector control across South America, particularly in epidemic contexts requiring the immediate reduction of vector populations and/or transmission. Unfortunately, the rapid emergence and spread of insecticide resistance across multiple vector species constitute a growing threat. Resistance is now widespread in South America, affecting major vector groups such as mosquitoes and triatomines [7,8]. Resistance to major insecticide classes—pyrethroids, organophosphates, and carbamates—has been documented across most countries, reflecting decades of sustained selection pressure from public health interventions, domestic insecticide use, and agricultural practices, and may be further exacerbated by environmental and climate change.

The most critical situation concerns *Aedes aegypti*, the primary dengue vector, in which multiple resistance to public health insecticides is widely reported [9]. This includes high levels of pyrethroid resistance and an increase in the frequency of well-characterized *kdr* mutations, with some Brazilian populations showing near fixation of resistant alleles [10]. These trends raise serious concerns about the operational efficacy of commonly used pyrethroids for vector control, including space spraying, long-lasting insecticide-treated nets (LLITNs), spatial repellents and indoor residual spraying. Increasing concern has also been raised regarding the tiger mosquito *Aedes albopictus*, an invasive and highly adaptable species [11] whose epidemiological importance is growing in the region. A recent systematic review highlights insecticide resistance in *Ae. albopictus* has been reported in several countries in Latin America and the Caribbean, although data remain limited and geographically uneven [12]. The study suggests that interventions targeting *Ae. aegypti* may exert indirect selection pressure on *Ae. albopictus*, potentially accelerating its adaptation to insecticides.

Resistance is also emerging in *Anopheles* species in malaria-endemic areas. Recent studies in the Amazon region reported increasing levels of pyrethroid resistance in *Anopheles darlingi* [13]. Although no *kdr* or *ace-1*^*R*^ mutations have been identified so far in this species [14], genomic analyses of more than 1,000 genomes suggest the involvement of detoxification enzymes, particularly cytochrome P450 monooxygenases, in pyrethroid resistance [15]. These developments raise important concerns about the long-term sustainability of malaria control strategies and their potential impact on elimination efforts.

Despite advances in understanding vector resistance dynamics, major knowledge gaps persist. Beyond well-characterized *kdr* mutations, molecular markers of resistance remain poorly described for most vector species of public health importance. Resistance surveillance systems across South America are fragmented, with limited standardization of methodologies and uneven geographic coverage. Available data are strongly biased toward a few countries, particularly Brazil, while large areas of the continent lack routine monitoring [6]. These gaps hinder the development of evidence-based resistance management strategies and limit the capacity of national programs to respond effectively and in a timely manner. Enhanced regional coordination, cooperation, and political support are critical to develop and implement effective strategies to contain and manage insecticide resistance.

In this context, the WINSA (Worldwide Insecticide Resistance Network—South America) initiative was established to foster collaboration, harmonize surveillance schemes, and strengthen research and training efforts across the region [6]. The WINSA workshop, held in Bogotá, Colombia, from 9 to 14 March, aimed to assess the current status of insecticide resistance in the 13 countries and territories of the region, identify major challenges and research priorities, and explore opportunities for coordinated regional action. It brought together key experts from academia and public health institutions to present the latest findings on vector resistance and to provide hands-on technical training on updated WHO procedures for monitoring insecticide resistance in mosquito populations.

This proceedings paper summarizes the main findings and recommendations arising from the workshop, intending to strengthen South American responses to insecticide resistance in disease vectors.

## Welcoming addresses

The opening session featured welcome remarks by Dr. Mauricio Torres-Tovar (Director of Public Health, UNAL) and Dr. Martha Quiñones (Professor, Department of Public Health, UNAL), who stressed the importance of initiatives like the WINSA network for strengthening public health and advancing research on vector control and insecticide resistance in Latin America. They emphasized the importance of strong institutional collaboration, including both North–South and South–South partnerships. The session then featured a presentation by Dr. Ademir Martins and Dr. Vincent Corbel, who introduced the WINSA network, outlining its origins, core objectives, and its role in fostering regional cooperation for the surveillance and management of insecticide resistance.

## Introduction conferences

The opening sessions featured three keynote lectures on the challenges and future directions for managing insecticide resistance across multiple scales, drawing on experiences from Brazil, Colombia, and Mexico. Collectively, these contributions highlighted key operational and scientific challenges, identified strategic priorities, and underscored emerging trends in the prevention and control of insecticide resistance in disease vectors.

**Dr. Martha Liliana Ahumada (Instituto Nacional de Salud, Bogotá, Colombia)** presented a study on the relationship between insecticide resistance and vector competence (VC) in *Ae. aegypti.* Vector competence refers to the ability of mosquitoes to acquire, maintain, and transmit pathogens such as dengue, Zika, and chikungunya viruses [14]. In Colombia, resistance to lambda-cyhalothrin in *Ae. aegypti* is associated with the *kdr* mutations V1016I and F1534C. A significant correlation was observed between the frequency of the 1016I allele and the 50% lethal concentration (LC50) of this insecticide. To assess the impact of resistance on VC, three strains of *Ae. aegypti* (susceptible, resistant, and highly resistant) were experimentally infected with DENV-2, ZIKV, and CHIKV. A total of 857 mosquitoes were analyzed using RT-PCR to estimate infection and dissemination rates. Results showed that the highly resistant strain exhibited higher dissemination metrics (Dissemination Infection Rate and Dissemination Efficiency), particularly for ZIKV, suggesting a potential link between insecticide resistance and increased viral transmission. However, no effect of *kdr* mutations on VC was observed across all viruses evaluated. Overall, the findings highlight complex interactions between mosquito resistance status and virus-specific dynamics, suggesting that insecticide resistance may, in some cases, enhance transmission potential, particularly for the Zika virus.

**Dr. Ademir J. Martins (FIOCRUZ-IOC, Brazil)** discussed Brazil’s comprehensive Insecticide Resistance Monitoring (IRM) program, operational for over 25 years and initiated through the MoReNAa network [16]. Against the backdrop of an increasing arbovirus burden in Brazil, the program follows a strategic framework aligned with WHO and PAHO guidelines, using laboratory and field-simulated bioassays to evaluate the efficacy of both larvicides and adulticides. A key component of this initiative is the integration of classical bioassays with molecular surveillance to identify resistance mechanisms, specifically metabolic detoxification enzymes and target-site alterations such as *kdr* mutations. Long-term monitoring has detected widespread resistance to traditional compounds like temephos and deltamethrin, with recent data confirming high frequencies of *kdr* alleles across nearly all evaluated Brazilian localities [10]. While newer larvicides such as spinosad still demonstrate full susceptibility, recent evaluations point to the possible emergence of resistance to novel adulticide formulations combining pyrethroids with clothianidin or imidacloprid [17]. Looking forward, the program emphasizes the need to maintain genetic biobanks for molecular surveillance and to expand resistance monitoring to other important vectors, such as *Anopheles darlingi*, triatomines and phlebotomines, while ensuring that novel control tools like *Wolbachia* are carefully matched to the genetic background of local populations.

**Dr. Adriana E. Flores (Universidad Autónoma de Nuevo León, Mexico**) delivered a keynote lecture on the evolution, current challenges, and future perspectives of chemical vector control. The lecture highlighted the global reliance on a limited number of insecticide classes for dengue vector control, noting that pyrethroids account for 94.4% of insecticides used for adult mosquito control through indoor residual spraying (IRS) and space spraying, whereas organophosphates, primarily temephos, account for 85.2% of larvicides [18]. This heavy reliance on a limited number of chemical classes creates strong and sustained selection pressure on vector populations. The lecture reviewed the major mechanisms of insecticide resistance reported in mosquitoes, including target-site mutations such as kdr mutations in the voltage-gated sodium channel, the G119S mutation in acetylcholinesterase (ACE1), and the A302S mutation in the GABA receptor (Rdl), as well as metabolic resistance mediated by detoxification enzymes, including esterases, glutathione S-transferases, and cytochrome P450 monooxygenases (CYP450). The lecture also emphasized that innovation in vector control should extend beyond the development of new insecticides, a process that requires substantial investment (approximately USD 300 million) and up to 10 years of development [19]. Emerging tools discussed during the lecture included long-lasting microencapsulated formulations, dual-mode-of-action products, attractive toxic sugar baits (ATSBs), spatial emanators, and endectocides. Finally, the lecture highlighted four key components of insecticide resistance management: (i) routine susceptibility monitoring and early detection, (ii) correct insecticide dosing and application, (iii) rotation of insecticides with different modes of action, and (iv) integration of chemical and non-chemical approaches within an integrated vector management framework.

## The WINSA initiative: A research network to tackle insecticide resistance in insect vectors in South America

**Dr Vincent Corbel (IRD, France, FIOCRUZ, Brazil**) continued the session by presenting the mission of the South American Research Network for the Surveillance and Control of Insecticide Resistance in Arthropod Vectors (WINSA). Established in 2024 by the IRD and FIOCRUZ, with support from the U.S. CDC VecNet initiative, WINSA brings together leading technical experts from research institutions across 13 countries and territories in South America, as well as the United States and France (WIN South America - WIN IRD). The network aims to foster collaboration and information exchange, identify research gaps and priorities, strengthen technical capacity in insecticide resistance monitoring, and support national and regional programmes addressing vector resistance [7]. Since its inception, WINSA has published research papers addressing key knowledge gaps related to vector resistance in South America [5,7,12] and has organized two technical workshops focused on biological and molecular methods for detecting resistance in mosquitoes (Rio de Janeiro, 2025; Colombia, 2026). The network has also supported five inter-country scientific exchanges, enabling staff to acquire new skills, strengthen research collaborations, and promote career development. In addition, WINSA is currently leading a joint research trial funded by the American Mosquito Control Association (AMCA) to address critical knowledge gaps in chemical control interventions for Culicoides vectors. The network is gaining increasing recognition for its role in addressing insecticide resistance threats, with the ultimate goal of contributing to the reduction and elimination of vector-borne diseases in South America. An overview of the scientific and technical contributions presented during the workshop, including the countries and institutions represented, the main vector groups or topics addressed, key findings, and the status or source of the information presented, is provided in Table 1.

**Table 1:** Overview of scientific and technical contributions presented during the 2026 WINSA workshop in Bogotá.

Country/territory	Institution	Main vector/topic	Contribution presented	Source/status
Colombia	Instituto Nacional de Salud	*Ae. aegypti*	Insecticide resistance, *kdr* mutations and vector competence	Workshop presentation/supporting published evidence
Brazil	FIOCRUZ-IOC	*Ae. aegypti* and other vectors	National insecticide-resistance monitoring and integration of phenotypic and molecular surveillance	Published/ongoing national surveillance
Mexico	Universidad Autónoma de Nuevo León	Vector control	Evolution and future perspectives of chemical vector control and resistance management	Workshop keynote/published evidence
Argentina	CIPEIN	*Ae. aegypti*	Pyrethroid resistance and chemical-control challenges	Published
Suriname	AdeKUS	*Ae. aegypti*	Resistance to λ-cyhalothrin and first evidence of malathion resistance	Published/reported study
Ecuador	UBA-GADMQ	*Ae. aegypti*	Longitudinal resistance surveillance and entomovirological monitoring	Published/ongoing surveillance
Brazil	FIOCRUZ-IOC	*Ae. aegypti*	National susceptibility to spinosad	Published
Peru	Instituto Nacional de Salud	*Ae. aegypti*	Nationwide deltamethrin-resistance intensity and *kdr* distribution	Workshop presentation/ongoing study
Venezuela	Instituto de Altos Estudios	*Anopheles* spp.	Insecticide susceptibility and metabolic resistance in malaria vectors	Workshop presentation/supporting published evidence
Uruguay	Ministerio de Salud Pública	*Ae. aegypti*	Phenotypic and molecular monitoring of pyrethroid resistance	Preliminary/ongoing study
Chile	Universidad Metropolitana/ Universidad de Valparaíso	*Ae. aegypti*	Detection and spatial distribution of *kdr* mutations in Rapa Nui	Workshop presentation/ongoing study
French Guiana	Institut Pasteur de la Guyane	*Ae. aegypti*, *An. darlingi*	Resistance surveillance in arbovirus and malaria vectors	Published/ongoing surveillance
Bolivia	INLASA	*Aedes*, *Anopheles* and triatomines	Resistance patterns and use of surveillance data for resistance management	Workshop presentation/national surveillance
Paraguay	Universidad Nacional de Asunción	*Ae. aegypti*	Residual efficacy of microencapsulated insecticidal paints	Workshop presentation
Colombia	Universidad Nacional de Colombia	Multiple mosquito genera	Transfluthrin spatial repellents for reducing human–vector contact	Workshop presentation
Guyana	Ministry of Health	Mosquito surveillance	Vector diversity and development of insecticide-resistance monitoring	Ongoing surveillance

The table summarizes contributions presented during the workshop and should not be interpreted as a comprehensive inventory of insecticide-resistance research or vector-control activities in each country.

## Country presentations to review challenges and knowledge gaps in vector surveillance and control in South America

The country presentations summarized below reflect the scientific, surveillance, and operational information shared by participants during the workshop and were intended to identify major resistance patterns, knowledge gaps, and technical challenges across the region. They should not be interpreted as comprehensive inventories of national vector-control programmes, policies, or interventions, which would require systematic consultation with the competent national and local health authorities.

**Dr. Laura Harburguer (CIPEN, Argentina)** reviewed the historical evolution and current challenges of chemical control strategies against *Ae. aegypti* in Argentina. This mosquito was successfully eradicated in the 1960s through coordinated continental campaigns but re-emerged in the 1980s, rapidly spreading across the country, including temperate regions previously considered unsuitable. Its expansion has been driven by human activity and environmental adaptation, with genetic studies suggesting multiple reintroduction routes from neighboring countries. Since reinfestation, vector control has relied mainly on chemical interventions, including pyrethroid-based adulticides and larvicides. While these tools have reduced mosquito populations during outbreaks, their effectiveness has been limited by operational and environmental constraints. A major challenge is the widespread development of insecticide resistance, particularly to pyrethroids, associated with *kdr* mutations such as V1016L, F1534C, and V410L [20,21]. This situation underscores the need for resistance management strategies, including rotation with alternative compounds such as organophosphates. Research institutions like CIPEN have contributed to evaluating and improving vector control tools. However, sustainable control of *Ae. aegypti* will require integrated strategies combining chemical, environmental, and social approaches, supported by stronger surveillance, community participation, and intersectoral collaboration.

**Dr. Kartika Ramoutar (AdeKUS, Suriname)** presented results from a recent study in Suriname providing new insights into insecticide resistance in *Ae. aegypti*. Between March 2023 and August 2025, mosquito populations from three districts were evaluated using CDC bottle bioassays and molecular characterization of *kdr* mutations. Bioassays revealed widespread resistance to λ‑cyhalothrin across all study sites and, for the first time in the country, resistance to malathion, contrasting with earlier reports of full susceptibility. Genotyping of three loci associated with resistance (*kdr* 410, 1016, and 1534) identified multiple tri-locus genotypes with high frequencies of resistant alleles [22]. These findings indicate established resistance to λ‑cyhalothrin and emerging malathion resistance. Similar trends, combining pyrethroid resistance, *kdr* mutations, and reduced susceptibility to organophosphates, have been documented in neighboring regions, highlighting the importance of ongoing surveillance, integration of phenotypic and molecular approaches, and strengthened regional cooperation to support evidence-based vector control strategies.

**MSc. Diego Morales (UBA-GADMQ, Ecuador)** presented results from a seven-year longitudinal surveillance program, providing new insights into insecticide resistance dynamics in *Ae. aegypti* in Ecuador. Between 2019 and 2025, mosquito populations from multiple localities across the Coastal, Andean, and Amazon regions were evaluated using bioassays and molecular characterization of resistance-associated mutations. Bioassays revealed a progressive, widespread increase in resistance to deltamethrin, with nationwide loss of efficacy, while malathion, previously broadly effective in 2019, showed emerging resistance by 2025, especially in Coastal and Amazon regions [23]. Resistance to temephos was also documented in areas previously considered susceptible, indicating geographic expansion. Molecular analyses confirmed the presence of *kdr* mutations (V1016I, V410L, and F1534C), validating established mechanisms of pyrethroid resistance. Complementary entomovirological surveillance detected active circulation of dengue serotypes DENV-1 and DENV-2 in field-collected mosquitoes [24]. These findings highlight that integrating insecticide resistance monitoring with viral surveillance is essential for guiding vector control strategies, optimizing insecticide use, and improving early detection of arbovirus transmission risk.

**Dr. Luciana Dias (FIOCRUZ, Brazil)** presented an assessment of *Ae. aegypti* susceptibility to spinosad in Brazil. Spinosad, a larvicide derived from soil bacteria with a distinct mode of action targeting the insect nervous system, was introduced in Brazil after previous laboratory evaluations demonstrated high susceptibility in different mosquito populations [25]. The monitoring approach involved the collection of 46 field populations across the country using ovitraps. This was followed by laboratory rearing to the F1 generation and standardized bioassays conducted according to World Health Organization guidelines. The discriminatory concentration (DC) was established through dose–response assays using the susceptible Rockefeller strain and subsequently applied to field populations. Results showed mortality rates between 99% and 100% to the DC, indicating that all tested populations remained susceptible to spinosad according to WHO criteria [17]. Overall, these findings reinforce the effectiveness of spinosad as an important vector control tool, owing to its distinct mode of action, while highlighting the need for rational use and ongoing monitoring to preserve its efficacy over time.

**Dr. Miriam Palomino Salcedo (Instituto Nacional de Salud, Peru)** presented the results of a nationwide monitoring study of 38 field populations across coastal, high jungle, and Amazonian regions, using WHO bioassays at standard DC, as well as at 5× and 10× DC to examine the resistance intensity. All populations were confirmed resistant to deltamethrin, with mortality rates well below the 98% susceptibility threshold. Most populations exhibited high-intensity resistance, particularly in the coastal and Amazonian regions, where mortality remained below 98% even at 10× DC. Molecular analyses revealed a high prevalence of *kdr* mutations (V410L, V1016L, and F1534C), with tri-locus resistant genotypes (R1R2, R1R1, and R2R2) accounting for more than 90% of observed genotypes nationwide. A significant correlation between *kdr* allele frequencies and resistance phenotypes was observed. Notably, resistance persists despite the discontinuation of pyrethroids in national control programs, suggesting ongoing selection from alternative sources such as domestic and agricultural insecticides. Knowing that Peru already exhibits widespread distribution of temephos resistance across the country [26], the authors underscore the urgent need to strengthen resistance surveillance and implement integrated vector management strategies, including the incorporation of innovative tools, in order to sustain the effectiveness of arbovirus control programs.

**Dr. Luisa Elena Figueroa Acosta (Instituto de Altos Estudios, Venezuela)** presented findings on insecticide susceptibility in *Anopheles* populations from Margarita Island, Venezuela. *Anopheles aquasalis*, the main coastal malaria vector, was found alongside with *Anopheles albimanus*, *Anopheles benarrochi,* and *Anopheles nuneztovari*, with the latter two reported for the first time in the insular region. CDC bottle bioassays showed that *An. aquasalis* was susceptible to lambda-cyhalothrin but resistant to fenitrothion. Synergist assays with piperonyl butoxide and biochemical tests indicated metabolic resistance involving altered acetylcholinesterase, oxidases, and esterases, consistent with previous studies in Venezuela [27]. *Anopheles nuneztovari* remained susceptible to carbamates and pyrethroids but showed resistance to fenitrothion. These results provide the first evidence of insecticide resistance in *Anopheles spp*. from Nueva Esparta, Venezuela.

**Dr. Gabriela Willat (Ministerio de Salud Pública de Uruguay**) presented results from a monitoring study on insecticide resistance in *Ae. aegypti* in Uruguay, conducted at the Vector Laboratory, Academic Unity of Parasitology and Micology of the Instituto de Higiene, and the Ministry of Public Health. The study assessed populations from different regions of Uruguay by analyzing two well-known *kdr* mutations, V1016G and F1534C, together with phenotypic resistance using the CDC Bottle Bioassay. The integration of molecular and phenotypic approaches provided valuable information on the status of pyrethroid resistance in these Uruguayan populations of *Ae. aegypti*. These preliminary data had also been presented at the 2025 Academic Meeting of the Biomedical Research Program (ProInBio). This work represents the first combined characterization of pyrethroid resistance in Uruguay and highlights the importance of continuous resistance surveillance and integrated vector management strategies.

**Dr. Christian R. González (Universidad Metropolitana, Santiago, Chile)**, in collaboration with the Escuela de Medicina, Universidad de Valparaíso (Chile), presented a study on insecticide resistance in *Ae. aegypti* from Rapa Nui. Although *Ae. aegypti* has been present on Rapa Nui since 2000, its resistance profile had not previously been characterized. This study aimed to detect *kdr* mutations associated with pyrethroid resistance in this primary dengue vector. A total of 112 specimens collected across the island were analyzed using Sanger sequencing. The results showed a high frequency (75%) of the I1011M mutation, while other common mutations (V410L, V1016I, F1534C) were not detected. Spatial analyses, combining statistical models and supervised learning, revealed heterogeneous distribution patterns of the I1011M mutation, suggesting localized selection processes. This is the first report of *kdr* mutations in Rapa Nui and mainland Chile. These findings highlight the need to continue resistance monitoring programs, integrating both molecular and spatial approaches to improve vector control strategies.

**Dr. Jean Bernard Duchemin (Institut Pasteur of French Guiana)** presented an overview of the monitoring of insecticide resistance in arbovirus and malaria vectors in French Guiana, as conducted by the Institut Pasteur of French Guiana for local health authorities. The specific context of French Guiana, as a European territory in South America, has limited the range of chemical insecticides available, with pyrethroids being the only option for adult mosquito control for decades, targeting *Ae. aegypti* and *Anopheles darlingi*. This has led to high levels of resistance in *Ae. aegypti* [28], within increased esterase and P450 activities, associated with the *kdr* mutation V1016I. We also found almost fixed the *kdr* mutation F1534C, and a high frequency of double mutants at positions 1534/1016. More recently, genotyping at position 410 revealed a V410L allelic frequency of 97.5% and a frequency of 96.7% for double mutant individuals (410/1016) (*n* = 120). In 2025, susceptibility tests were conducted in the Cayenne area on *An. darlingi* showed 92% mortality with deltamethrin. This may be linked to strong signals of selection, likely driven by insecticide pressure, as highlighted in a large genomic study of *An. darlingi* in South America [15]. These results underline the need for strengthened regional collaboration on malaria vectors, as well as the development of innovative alternatives to chemical insecticides for *Ae. aegypti* control.

**Dr. Ronald López (INLASA, Bolivia)** opened his presentation by describing the historical development, organizational structure, and main divisions of the Entomology Laboratory at INLASA. He then presented an overview of malaria vector distribution in Bolivia, along with collection methods, bioassays, and major findings. Results revealed the presence of pyrethroid resistance in certain regions, primarily driven by metabolic mechanisms. Notably, switching vector control interventions to bendiocarb led to a reversion of resistance, restoring susceptibility to pyrethroids. Subsequently, he reported on studies of *Ae. aegypti* populations, showing widespread resistance to pyrethroids across Bolivia, likely associated with genetic mechanisms, while susceptibility to organophosphates remains. Finally, he presented findings on vectors of Chagas disease, indicating resistance to pyrethroids but maintained susceptibility to carbamates. Overall, the presentation demonstrated how these data support evidence-based decision-making by the Bolivian Ministry of Health for the management of insecticide resistance in vector control programs.

The session finished with two complementary presentations exploring innovative tools to reduce human–vector contact and improve vector control in contexts of increasing insecticide resistance.

**Dr. Nilsa González Brítez (Universidad Nacional de Asunción, Paraguay)** presented a study evaluating the residual efficacy and repellent effect of microencapsulated insecticidal paints against *Ae. aegypti* in San Lorenzo, Paraguay. Four formulations were tested on raw concrete, painted concrete, and wood following WHO protocols: (A) pirimiphos-methyl + pyriproxyfen, (B) transfluthrin, (C) chlorfenapyr + pyriproxyfen, and (D) propoxur. A total of 1,200 unfed females were exposed at intervals from 7 to 270 days to assess mortality and irritant effects. Results showed high initial mortality that declined over time, with variations depending on the surface and formulation. Propoxur (D) demonstrated the strongest residual efficacy, whereas the chlorfenapyr/pyriproxyfen mixture (C) showed more variable mortality but stronger irritant effects. In conclusion, propoxur appears to be the most suitable option for sustained control, while chlorfenapyr could be used as a complementary tool for its irritant properties, particularly in resistance settings. Continuous monitoring of efficacy remains essential to guide adaptive vector control strategies.

**Dr. Martha Quiñones (Universidad Nacional de Colombia, Colombia**) presented a study evaluating the effect of the volatile pyrethroid transfluthrin applied to hessian strips and mats to reduce human–vector contact in the peridomestic areas of households in Guapi, Cauca, Colombia. Previous studies in Africa have shown that outdoor spatial repellents can significantly reduce human–vector contact [29], but evidence from Latin America has been limited. In this study, mosquito collections were conducted from 6:00 to 10:00 p.m. in peridomestic areas before and after the installation of hessian strips and mats impregnated with transfluthrin (0.38%). Several mosquito species were collected, including the malaria vectors *An. albimanus* (12%) and *Anopheles neivai* (2%), the arbovirus vectors *Ae. aegypti* (4.7%) and *Ae. albopictus* (18.6%), and *Culex quinquefasciatus* (57%), which was present at high densities and represents an important biting nuisance. The intervention resulted in a reduction in human–vector contact of 84.2% for *Aedes* mosquitoes, 92.9% for *Culex,* and 95.6% for *Anopheles*, with protective effects lasting up to 12 weeks after installation. These findings suggest that transfluthrin-impregnated strips and mats used outdoors could serve as an effective complementary tool for vector control, helping to reduce mosquito bites and the risk of transmission of diseases such as malaria, dengue, and Zika. Further studies are needed across larger areas, diverse ecological settings, and different vector species to confirm and expand these results.

**Ms Tashanna Bowman (Vector Control Services, Ministry of Health, Guyana)** presented an overview of the current vector surveillance activities in Guyana. Her presentation highlighted findings from a survey of mosquito species conducted in selected communities between May and September 2025. The study documented the first records of *Mansonia indubitans* and *Culex adamesi* in the country and confirmed the presence of *Ae. albopictus* in Guyana. Future directions for surveillance in support of disease control and elimination efforts were also discussed. These included plans to expand surveillance to other vectors of medical importance and to initiate insecticide resistance monitoring programs for both malaria and dengue vectors. Together, these initiatives represent an important step toward strengthening national entomological surveillance capacity and promoting evidence-based vector control interventions in Guyana.

## Gaps to fill for strengthening insecticide resistance monitoring and management in South America

### 1. Understanding resistance mechanisms: A key to effective surveillance and management

During the workshop, all participants emphasized that understanding insecticide resistance (IR) mechanisms is a fundamental pillar for the success of entomological surveillance and vector control programs. The main physiological adaptations that confer resistance in mosquitoes, include reduced insecticide penetration through cuticle thickening, metabolic resistance mediated by detoxification enzymes (such as P450s, GSTs, and CCEs), and target-site modifications, particularly *kdr* mutations in the voltage-gated sodium channel. Data on *Ae. aegypti* in Brazil and across the Americas illustrate adaptive convergence, whereby identical *kdr* mutations arise independently in distinct populations [30]. Monitoring efforts have revealed substantial spatial variation in allelic frequencies, including a high prevalence of “rare” genotypes in specific regions such as Oiapoque (French-Brasilain border), highlighting the importance of high-resolution molecular surveillance to map the spread of resistant vectors over time [31]. The integration of genetic markers enables the detection of resistance mechanisms before they become phenotypically apparent in standard bioassays. This is critical for identifying “tipping points” in resistance evolution and for guiding insecticide selection to minimize cross-resistance [32]. Furthermore, advanced genomic approaches, including genome-wide association studies (GWAS), transcriptomics, and next-generation sequencing (NGS), offer powerful tools to identify novel resistance mechanisms, trace routes of introduction and migration, and monitor the evolutionary dynamics of resistance. The recommendations for a timely response to resistance are to: (i) identify and validate relevant genetic markers of resistance, including metabolic mechanisms; (ii) strengthen diagnostic capacity by integrating molecular tools into routine surveillance systems; and (iii) establish a regional surveillance network to detect resistance hotspots and support the implementation of coordinated resistance management strategies.

### 2. Integrating insecticide resistance data into national vector surveillance systems for evidence-based decision-making

Despite recent advances in methodologies and protocols for monitoring insecticide resistance in vectors [33,34], there remains a lack of operational guidance on how to use resistance data to inform decision-making. Participants therefore proposed the development of an integrated framework to strengthen resistance monitoring and management in *Aedes* mosquitoes, which currently represent a major challenge for vector control programs (Fig 1).

**Fig 1:**
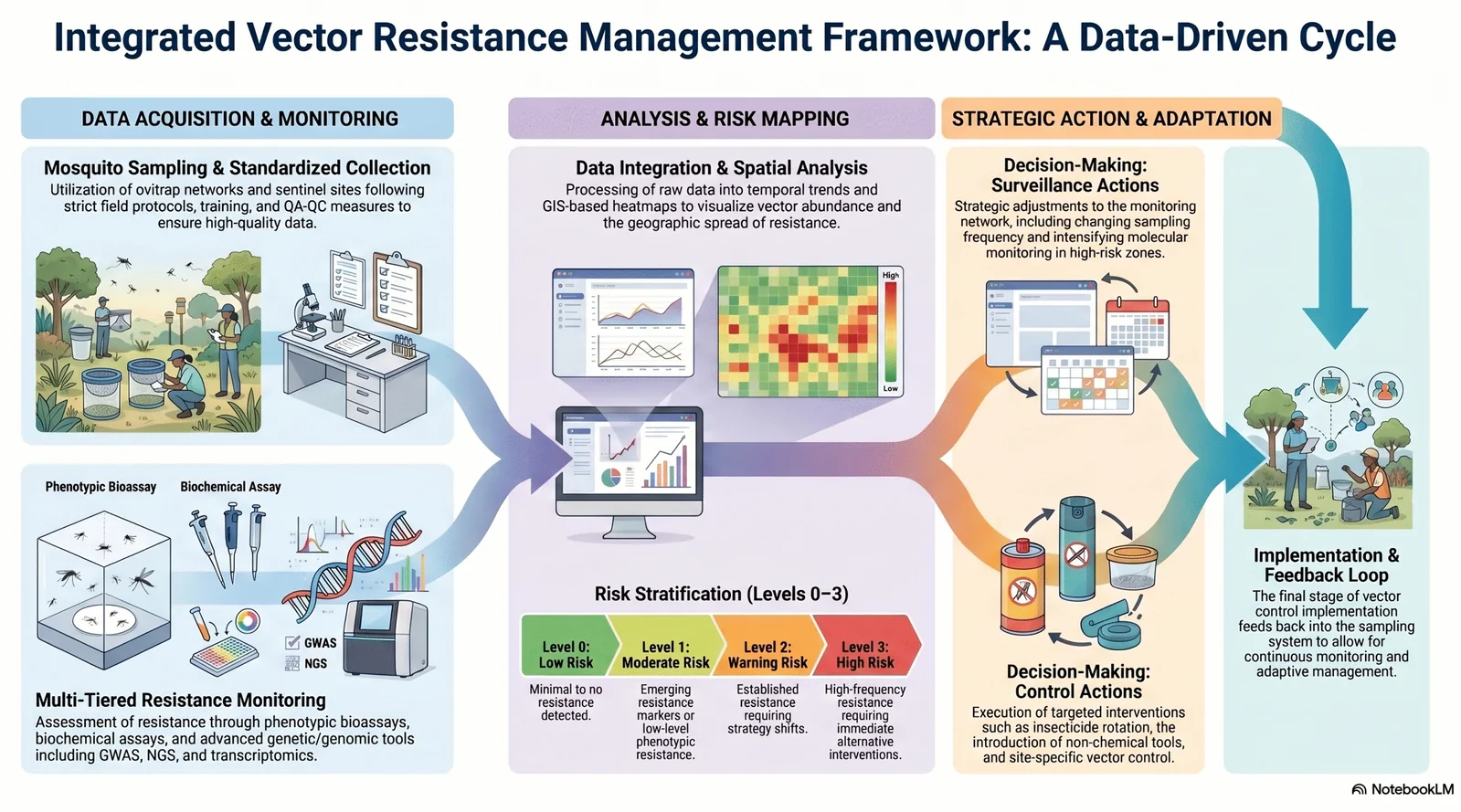
Integrated Vector Resistance management framework: a data-driven cycle.

Although the framework presented here was developed primarily in the context of *Aedes* resistance surveillance, WINSA encompasses insecticide resistance across multiple arthropod vectors of public health importance. The network does not propose a single operational resistance-management scheme uniformly applicable across vector groups, insecticides, or diseases. Instead, it promotes common principles of standardized resistance monitoring, characterization of resistance mechanisms, quality assurance, data sharing, capacity building, and evidence-based interpretation. Their operational application should remain vector- and context-specific, considering differences in vector biology and ecology, disease epidemiology, insecticide exposure, available control tools, and national surveillance and control systems. These general principles may therefore provide a basis for future adaptation to *Anopheles*, triatomines, flebotomines and other medically important vectors.

The first step is to ensure adequate and standardized mosquito collection methods. Standardization is essential to generate reliable and comparable data that can effectively guide vector surveillance and control decisions. A good example comes from Brazil’s national strategy for monitoring *Ae. aegypti* presence, abundance, and insecticide resistance. This system, developed by the Ministry of Health, is based on territorial planning and standardized protocols [35]. It includes the selection of municipalities based on epidemiological, demographic, and operational criteria, combined with ovitrap-based surveillance and grid-based georeferencing to ensure spatial representativeness and comparability. Sampling designs are adapted to local contexts while maintaining analytical robustness. The program is institutionally structured across federal, state, and municipal levels, with laboratory analyses currently conducted by FIOCRUZ [35,36]. Its effectiveness relies on strong coordination, standardized procedures, rigorous quality control, and continuous training of field teams through partnerships between national and local stakeholders.

The second step is to shift from passive resistance monitoring to an operational, decision-oriented surveillance system that provides graduated, context-specific responses based on available evidence. In brief, the approach relies on periodic monitoring of insecticide resistance at the population level in predefined sites, using appropriate biological, molecular, and/or biochemical methods, and on stratifying resistance risk at the territorial level (0–3) to guide surveillance and vector control actions (Fig 1). This stratification linking surveillance outputs to vector control decision-making enables timely adjustments such as insecticide rotation or replacement strategies. This concept, initially developed for France and its overseas territories [37] and aligned with WHO standards and methodologies [33], can be readily adapted to South America. However, its implementation will require strong regional coordination and shared data platforms to enable the early detection of resistance trends.

## How can the WINSA network strengthen insecticide resistance monitoring and management in the region?

As discussed above, strengthening insecticide resistance monitoring and management in South America will require a coordinated and strategic approach, based on research, capacity building, networking, and expertise [7]. By bringing together entomology research institutions and public-health partners across the region, WINSA provides a collaborative scientific and technical platform to address knowledge gaps in vector surveillance and insecticide resistance. The network can support the harmonization of methodologies, quality assurance, data and expertise sharing, collaborative research, and the development of technical capacity across participating countries or institutions. Greater methodological harmonization can improve data comparability and facilitate regional analyses of spatial and temporal trends in insecticide resistance, while advances in genomic surveillance and spatial modeling may further strengthen the interpretation of resistance patterns.

WINSA’s role should nevertheless be distinguished from that of the institutions responsible for operational vector control. The network does not define national vector-control policies or determine the interventions implemented by participating countries. Research institutions and laboratories can contribute to generating, validating, and interpreting resistance data and to translating these findings into technical evidence for decision-making. Decisions regarding insecticide selection and rotation, procurement, deployment of interventions, and other operational responses remain the responsibility of the figtent national and local health authorities, according to each country’s governance structure, epidemiological context, and available control tools. WINSA can facilitate sustained dialogue among these stakeholders and support the translation of scientific evidence into locally appropriate resistance-management decisions.

## Conclusion

Insecticide resistance has become a major threat to the effectiveness and sustainability of vector control strategies in South America, undermining efforts to prevent arboviral diseases such as dengue, yellow fever, chikungunya and Zika, as well as parasitosis such as malaria, Chagas disease, and leishmaniasis. Discussions during the WINSA workshop highlighted the widespread distribution of resistance across multiple vector species, the increasing complexity of underlying resistance mechanisms, and substantial disparities in surveillance capacity among countries. In this context, strengthening insecticide resistance surveillance and management is essential to preserve the efficacy of existing control tools and reduce the burden of vector-borne diseases. A coordinated regional approach will be critical to achieving sustainable vector control and advancing health-related Sustainable Development Goals in South America.
